# An unexpected cellular fountain of youth: platelets provide factors rejuvenating brain functions

**DOI:** 10.1038/s41392-023-01716-w

**Published:** 2023-12-29

**Authors:** Harald F. Langer

**Affiliations:** 1 Department of Cardiology, Angiology, Haemostaseology and Medical Intensive Care, University Medical Centre Mannheim, Medical Faculty Mannheim, Heidelberg University, Mannheim, Germany; 2 German Centre for Cardiovascular Research (DZHK), Partner Site Heidelberg/Mannheim, Mannheim, Germany; 3 European Center for Angioscience, Medical Faculty Mannheim, Heidelberg University, Mannheim, Germany; 4 Cardiovascular systems biology, Medical Clinic I, University Heart Center Lübeck, Lübeck, Germany

**Keywords:** Neurodevelopmental disorders, Innate immunity

In a recent manuscript published in *Nature*, Schroer et al. discovered that platelets and in particular platelet derived PF-4 (CXCL4) are a novel factor, which can rejuvenate the mammalian neuronal system.^1^ These findings have the potential to trigger an entire new field of research opening new platelet-derived candidate effector molecules for supporting regeneration.

Platelets (also referred to as thrombocytes) are a-nuclear small blood cells, which derive from a common myeloid progenitor cell within the bone marrow and are constantly released from megakaryocytes into the blood circulation throughout our lives. One megakaryocyte can release up to 3000 platelets, their half-life within the circulation is 8 to 9 days and “defect” cells are removed particularly by the spleen but also by other organs. The original function of platelets is to provide provisional closure of vascular and tissue breaches and initiate repair mechanisms. Under pathophysiological conditions, however, platelet activation can be detrimental, as they have a supervillain power—the high-speed assembly of intravascular thrombi causing devastating diseases such as stroke or myocardial infarction. Furthermore, platelets are cellular chemical and biological messengers, as they store an array of highly active substances, which they may release very rapidly in a directed or a more unselected fashion depending on the surrounding molecular milieu and functional context.

Besides the established “classical” platelet functions, accumulating evidence draws a broader picture for their relevance of remove of in various settings of development, physiology and disease. For instance, their relevance has been thoroughly demonstrated for prevention of infections,^2^ tailoring the degree of revascularization of tissue ischemia, induction of tissue-remodelling processes such as apoptosis^3^ or the aggravation of NFAD – only to mention some. The elegant study by Schroer *et al*. now adds a fascinating new aspect to how platelets surprise researchers of what they are capable of.

During platelet activation, PF-4—and many other very active factors—is released from platelets locally or systemically. Originally, the cytokine PF-4 (alternative term: chemokine (C-X-C motif) ligand 4 (CXCL4)) belonging to the CXC chemokine family and binding to its canonical receptor CXCR3, was mainly investigated in the context of thrombosis. Strong attention was drawn to the molecule with the discovery that a specific PF4/heparin complex represents the HIT antigen.

In the discussed recent paper by Schroer et al., the authors characterize an unexpected rejuvenating effect of platelets and platelet-derived factors for the ageing brain. In particular, the platelet factor 4 (PF-4)—chemokine receptor CXCR3 axis was a central molecular pathway mediating cellular, molecular and cognitive benefits for the aged brain by ameliorating inflammation and rescuing cognition. The starting point and trigger for their studies was the observation from parabiosis experiments for the purpose of systemic rejuvenating interventions, whereby blood plasma preparations derived from young or exercised mice was capable of rejuvenating the aged brain.^4^ The authors deliberately checked on the important consideration that plasma preparations may contain relevant cellular components—for instance platelets—beyond the soluble factors previously made accountable for the observed effects on ageing.

It is very much appreciated that a broad approach by RNA-seq analysis was used by the authors to decipher the molecular keys changing in ageing brains as elicited by transfusing a young platelet fraction isolated from the blood, which yielded more than 600 differentially expressed genes. The fact that central topics such as development of the nervous system and immune regulation were spawned by the comparative Gene Ontology (GO) analysis promises us fascinating future research in this new scientific avenue. Not surprisingly, application of young but not old platelets reduced neuro-inflammation represented by a reduction of inflammatory microglia cells or a decrease in the complement activation protein C1q. Future in depth studies will have to address further potential effects on other pro (or anti-) inflammatory cells and mediators. Speaking of the complement system as a central part of innate immunity, the other pathways of complement activation not addressed in this study may be analyzed as well. This would make sense as published data suggest that activation of the complement system can trigger the release of PF-4 from platelets and subsequently anti-angiogenic effects.^5^ As one might assume that newly built vessels are beneficial for the brain, this observation at first sight seems controversial. However, the finding that PF-4 cannot cross the blood-brain barrier (using a tagged molecule PF-4 molecule) could explain that there was no negative effect of PF-4 in reducing supply of the brain by less angiogenesis.

Based on their results that PF-4 is more prevalent in “the young setting”, the authors chose the cytokine as a potential mediating mechanism. Using a pharmacological intervention strategy administering recombinant PF-4 and mouse models with a global knockout for PF-4 it was then demonstrated that systemic administration of PF4 can dial down neuroinflammation in the aged brain, and absence of PF4 aggravates neuroinflammation. Corroborating common knowledge, PF-4 had a wide range of effects on the immune system also in the author’s experimental setting. Only to mention a few aspects, the known shift affecting the ratio of myeloid to lymphoid cells in ageing organisms, was remarkably reversed in aged mice by PF4 treatment. Gene signature analysis furthermore showed that individual myeloid or lymphoid cells were partially “turning younger” in PF4 treated aged mice. Based on the plethora of positive findings corroborating their hypothesis, the authors conclude with reason that platelet-derived “PF4 in part restores the cellular composition and molecular signature of the ageing peripheral immune system to a more youthful state”. Importantly, the authors applied several tests for cognition with injection of young platelets or adequate control arms including forced alternation Y maze, novel object recognition (NOR), contextual fear conditioning paradigms and radial arm water maze (RAWM). In these behavioural tests, administration of PF4 systemically enhanced the aged brains cognitive functions. In contrast, the absence of PF4 accelerated cognitive deterioration in an age-dependent manner. It has to be mentioned that the authors applied no platelet-specific approach such as testing improvements in neuronal function using a cre-lox mouse system. Thus, it cannot entirely be ruled out that other cell types connected to the PF-4—CXCR3 system are also at least in part mediating the observed effects. Furthermore, also the PF-4-dependent crosstalk of platelets with other cells—for example leukocytes—should be investigated in future experimental concepts.

**Fig. 1:**
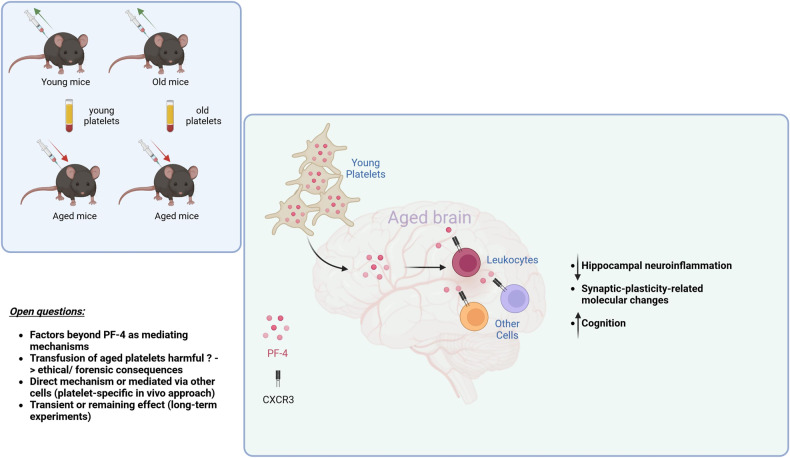
Rejuvenating actions of young platelets. In the presented manuscript, it was shown in an elegant fashion that re-injection of young platelets into aged mice can restore cognitive brain functions and ameliorate negative effects of neuronal inflammation in a systemic way but not within the central nervous system. Given that this observation is seminal, several open questions remain, such as identification of factors beyond PF-4 as a mediating factor, other involved mechanisms and ethical or direct clinical implications, respectively. This figure was created with BioRender.com

Based on data with tagged PF-4 indicating that the mechanism of action is rather in the periphery—involving the immune system—than in the central nervous system, the authors then applied mice deficient for the canonical PF-4 receptor CXCR-3. Along with the author´s prediction, the absence of CXCR-3 showed similar phenotypes to the lack of PF-4, while CXCR3 deficiency partly reverses the benefits on the aged hippocampus conferred by PF4 treatment (Fig. 1).

There are remaining open questions, for example concerning the duration of their experiments (injection of platelets 8 times over 24 days). It will be very curious to see, if the effects on the brain last over a long period, for instance at least over one year or even longer. It is also conceivable, that platelets or PF4 in platelet granules or PF4 in the patients´ plasma could be used as a diagnostic tool to deduce the risk for or determine the stage of neuroinflammatory diseases. Whether an improved genetic signature by the transfer of young platelets implies that the transfer of platelets from middle-aged, old or very old donors into very young, young or middle-aged patients is potentially harmful, has not been addressed. This question is important from a scientific point of view, but also has ethical and forensic implications (for example for transfusion medicine) and, thus, it has to be urgently addressed. Optimally, megakaryocytes or earlier platelet precursors could be modified within the bone marrow to constantly provide the organism with young platelets.

Overall, the work presents novel and very remove very exciting data opening entirely new scenarios, where platelets are cellular modulators of ageing processes for example by the release of immune modulating factors.
